# Fluorescence circadian imaging reveals a PDF-dependent transcriptional regulation of the *Drosophila* molecular clock

**DOI:** 10.1038/srep41560

**Published:** 2017-01-30

**Authors:** Virginie Sabado, Ludovic Vienne, José Manuel Nunes, Michael Rosbash, Emi Nagoshi

**Affiliations:** 1 Department of Genetics and Evolution, Sciences III, University of Geneva, 30 Quai Ernest-Ansermet, 1211 Geneva 4, Switzerland; 2 Laboratory of Anthropology, Genetics and Peopling History, Department of Genetics and Evolution, University of Geneva, 12 rue Gustave-Revilliod, 1211 Geneva 4, Switzerland; 3 Howard Hughes Medical Institute, National Center for Behavioral Genomics, Department of Biology, Brandeis University, 415 South Street, Waltham, Massachusetts 02451, USA.; 4 PRESTO, Japan Science and Technology Agency, 4-1-8 Honcho Kawaguchi, Saitama 332-0012, Japan

## Abstract

Circadian locomotor behaviour is controlled by a pacemaker circuit composed of clock-containing neurons. To interrogate the mechanistic relationship between the molecular clockwork and network communication critical to the operation of the *Drosophila* circadian pacemaker circuit, we established new fluorescent circadian reporters that permit single-cell recording of transcriptional and post-transcriptional rhythms in brain explants and cultured neurons. Live-imaging experiments combined with pharmacological and genetic manipulations demonstrate that the neuropeptide pigment-dispersing factor (PDF) amplifies the molecular rhythms via time-of-day- and activity-dependent upregulation of transcription from E-box-containing clock gene promoters within key pacemaker neurons. The effect of PDF on clock gene transcription and the known role of PDF in enhancing PER/TIM stability occur via independent pathways downstream of the PDF receptor, the former through a cAMP-independent mechanism and the latter through a cAMP-PKA dependent mechanism. These results confirm and extend the mechanistic understanding of the role of PDF in controlling the synchrony of the pacemaker neurons. More broadly, our results establish the utility of the new live-imaging tools for the study of molecular-neural interactions important for the operation of the circadian pacemaker circuit.

Orchestrated action of genetic programs and neuronal activity determines the emergent output of a neural circuit. The control of circadian behavioural rhythms is a prime example of this orchestration. Networks of dedicated pacemaker neurons, each containing a molecular clock, produce synchronized daily rhythms in gene expression and electrical activity, thereby driving coherent rhythmic behaviour. Owing to its numerically simpler nervous system and a battery of genetic tools, *Drosophila* offers an unusually powerful model to dissect the molecular and neural mechanisms of circadian behaviour.

The molecular clock of *Drosophila* consists of interlocked transcriptional-translational feedback loops, as in vertebrates. In the core feedback loop of the *Drosophila* clock, transcription factors CLOCK (CLK) and CYCLE (CYC) heterodimerize and activate transcription of *period (per*) and *timeless (tim*) through binding to their upstream and intronic E-boxes. Following the translation of *per* and *tim* mRNAs, PER and TIM translocate into the nucleus and inhibit CLK/CYC-mediated transcription. The CLK/CYC heterodimer also rhythmically activates transcription of a large number of output genes by binding rhythmically to their E-boxes, thereby controlling circadian rhythms in cellular functions^1^. Along with these transcriptional mechanisms, multiple post-transcriptional and post-translational mechanisms, particularly the post-translational modifications of PER that control its timed nuclear localization and degradation, are critical for the generation of circa 24-hr period rhythms^2^.

The circadian pacemaker circuit is composed of approximately 150 clock-containing neurons in the brain and controls the circadian locomotor rhythms of adult flies. Clock neurons are classified into 7 major subgroups: two classes of ventral lateral neurons (small-ventral lateral neurons, s-LNvs; large-ventral lateral neurons, l-LNvs), dorsal lateral neurons (LNds), lateral posterior neurons (LPNs), and three classes of dorsal neurons (DN1s, DN2s, and DN3s). Four out of five s-LNvs and all the l-LNvs express the neuropeptide Pigment-dispersing factor (PDF). The larval circadian circuit is composed of three classes of clock neurons (LNvs, DN1s and DN2s) and controls circadian light response behavior^3^,^4^. Larval PDF-positive LNvs are identical to the adult PDF-positive s-LNvs, which are the master pacemakers that control circuit synchrony and coordinate behavioural output^5^.

Previous studies have demonstrated the contribution of neuronal excitability and network interactions to rhythm generation in *Drosophila* pacemaker clock neurons. Similar to what is observed in the mammalian circadian pacemaker neurons located in the suprachiasmatic nuclei (SCN)^6^,^7^, continuous neuronal silencing and blockage of synaptic transmission do not abolish the molecular clockwork in *Drosophila* clock neurons, although these manipulations impair behavioural output^8^,^9^. A number of studies have shown that PDF plays critical roles in controlling synchrony in clock neurons and behavioural rhythms, analogous to the roles of vasoactive intestinal peptide (VIP) in the SCN^10^,^11^,^12^. PDF acts on multiple clock neuron subtypes through possibly diverse and cell type-specific mechanisms of action, e.g., controlling the phase, amplitude or pace of molecular clocks^13^,^14^,^15^,^16^,^17^,^18^,^19^. A recent study also demonstrated that PDF is required to control the phase of clock neuron output^20^. However, despite these advances in characterizing the role of PDF signalling in the circadian circuit, a mechanistic understanding of the precise interplay between the molecular clock and PDF signalling requires further investigation. Other neuropeptides and neurotransmitters are also involved in circuit synchronization and the coordination of behavioural output^21^,^22^; however, their impact on the molecular clockwork remains largely uncharacterized.

Precise mechanistic analysis of the interplay between network interactions and the molecular clockwork requires a system that enables the manipulation of network communication while simultaneously monitoring the dynamics of the molecular clock. To this end, here we develop two novel fluorescent reporters that permit real-time recording of the transcriptional and post-transcriptional machineries of the *Drosophila* clock in cultured neurons and whole brains. Using these new tools combined with genetic and pharmacological manipulations, we demonstrate that PDF enhances CLK/CYC-mediated transcription cell-autonomously at night via an activity-dependent, cAMP-independent mechanism. Our results confirm and extend the role of PDF in the molecular clock and more broadly demonstrate the utility of the novel live-imaging tools for investigating the molecular-neural interactions important for the operation of the circadian pacemaker circuit.

## Results

### Novel circadian reporters for live fluorescence imaging

**Figure 1:**
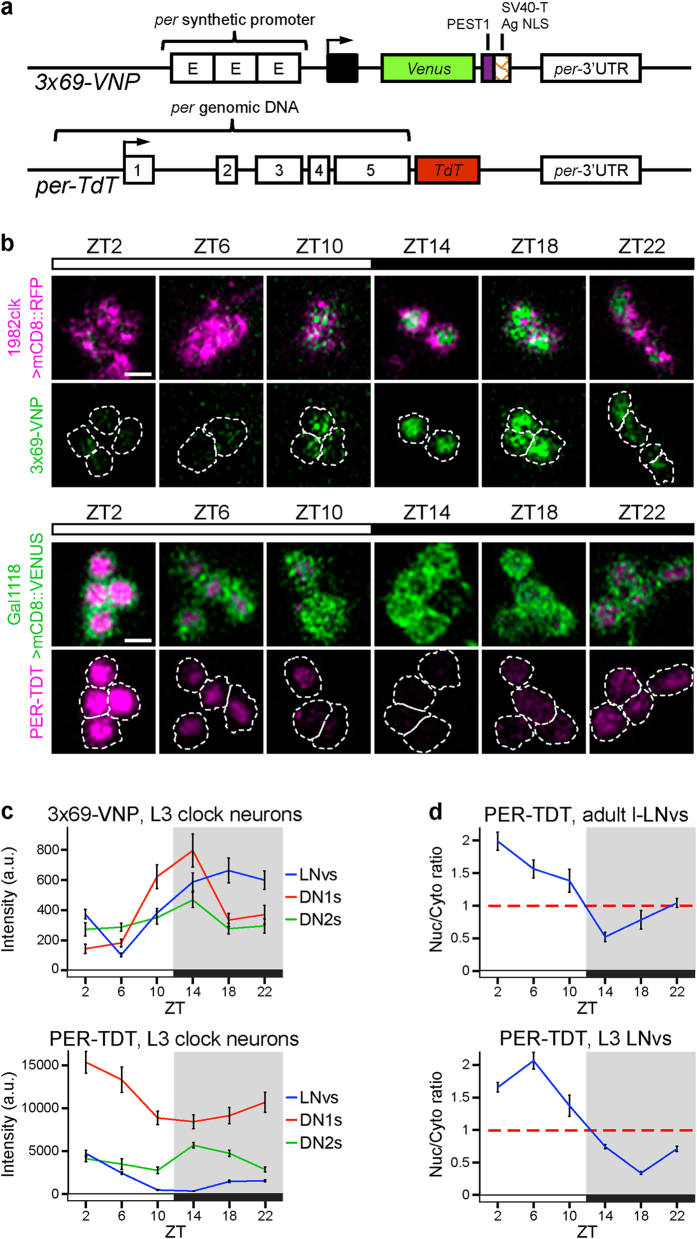
Novel fluorescent reporters of CLK/CYC-mediated transcription and PER protein oscillations. (**a**) Transcriptional (*3* × *69-VNP*) and PER protein (*per-TdT*) reporters. (**b**) 3 × 69-VNP and PER-TDT expression in third instar larval brains dissected every 4 hr in LD cycles. Representative confocal images of the LNvs are shown. Clock neurons in the *3* × *69-VNP* line were labelled by *1982clk-gal4, UAS-mCD8*::*RFP*. The LNvs in the *per-TdT* flies were labelled by *gal1118-GAL4, UAS-mCD8*::*VENUS*. Scale bars are 5 μm. (**c**) Quantification of the reporter fluorescence intensity in clock neurons. Mean ± SEM. A.U., arbitrary unit. A minimum of 30 LNvs, 17 DN1s and 11 DN2s were analysed at each timepoint. (**d**) Nuclear/cytoplasmic fluorescence ratio of PER-TDT in adult and larval LNvs.

Transcriptional activation from E-box-containing promoters and the feedback transcriptional inhibition by PER comprise the essential core loop of the molecular clock. Post-transcriptional regulatory features that control the stability, activity and subcellular localization of PER are also critical for the generation of circa 24-hr period rhythms^23^. Therefore, to monitor the functioning of the transcriptional and post-transcriptional machinery of the molecular clock at single-cell resolution, we generated two fluorescent reporters: one to monitor CLK/CYC transcriptional activity rhythms and the other to follow PER protein levels and localization (Fig. 1a).

The CLK/CYC transcriptional activity reporter *3* × *69-VNP* expresses short-lived yellow fluorescent protein fused to a nuclear localization signal (VENUS-NLS-PEST, VNP)^24^ under the control of three tandem repeats of the clock regulatory sequence (CRS), which is the E-box-containing 69-bp enhancer of *per*. The CRS is necessary and sufficient for *per*-like spatial and temporal transcriptional expression^25^, and the CRS trimer has been shown to drive CLK/CYC-dependent, high-intensity and high-amplitude circadian reporter expression *in vivo*^26^. The PER protein reporter *per-TdT* was modified from the previously established *BG-luc* reporter^27^ and was designed to express the N-terminal two-thirds of PER fused with tandem TOMATO (TdT) red fluorescent protein, under the control of *per* regulatory sequences and the 3′UTR. The N-terminal PER moiety harbours most of the known functional domains, including two PER-ARNT-SIM (PAS) domains and an NLS sequence, but it lacks the CLK/CYC inhibition domain and thus does not replace endogenous PER function^27^.

As expected, both molecular clock reporters were expressed rhythmically in adult and larval brain clock neurons (Fig. 1b,c, and S1a–d). A detailed analysis of the simple circadian circuit of third-instar larvae revealed that 3 × 69-VNP peaked between ZT14 (2 hr after lights off under a 12 hr:12 hr light-dark (LD) cycle) and ZT18, which corresponds approximately to the peak phases of *per* mRNA accumulation, CLK/CYC binding to *per* E-boxes, and genome-wide CLK/CYC binding^1^,^28^,^29^. PER-TDT peaked approximately 8 hr after the 3 × 69-VNP peak in the LNvs and DN1s, recapitulating the characteristic lag between *per* mRNA and protein accumulation^29^. PER-TDT rhythms in the DN2s were anti-phasic to the LNvs and DN1s, which is consistent with endogenous PER cycling^30^ (Fig. 1c and S1a). Furthermore, the cycling profile of PER-TDT nuclear-cytoplasmic localization was identical to that of PER (Fig. 1d)^31^. The cycling expression of both reporters and the rhythms in PER-TDT nuclear/cytoplasmic ratio were largely maintained in DD (Fig. S1c and d). These results establish that the *3* × *69-VNP* and *per-TdT* reporters provide reliable readouts of the molecular clockwork at a single-cell resolution; 3 × 69-VNP reports CLK/CYC-mediated transcriptional rhythms and also mimics *per* mRNA rhythms, whereas PER-TDT mimics PER protein oscillations.

**Figure 2:**
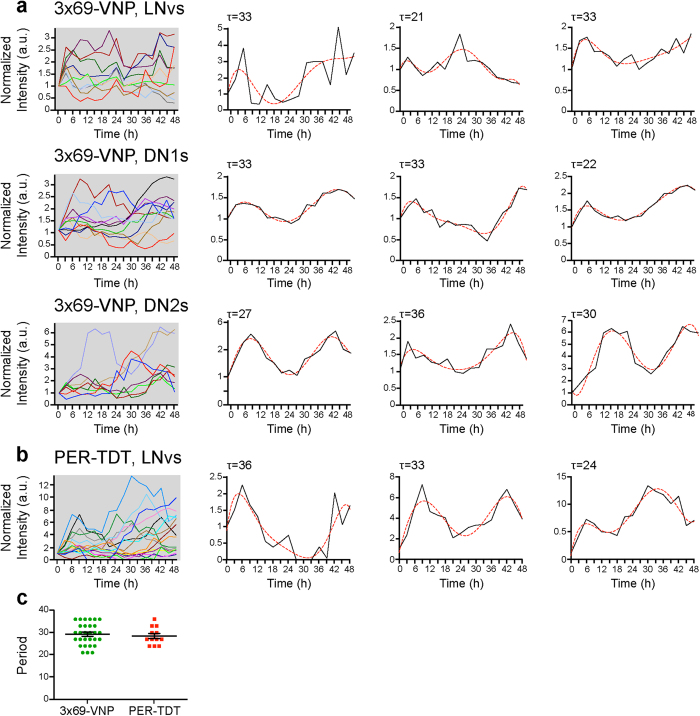
Fluorescent reporter expression in clock neurons in cultured larval brains. Whole-brain explants were prepared from LD-entrained larvae at ZT11 and then cultured and imaged in constant darkness (DD). Images were taken every 3** **hr for at least 48 hr from ZT18 of the last LD cycle. In *3* × *69-VNP* flies, all 3 larval clock neuron subtypes were labelled by driving expression of *UAS-mCD8*::*RFP* with *1982clk-gal4* and analysed. PER-TDT expression was monitored only in the LNvs labelled with *gal1118, UAS-mCD8*::*Venus* due to the technical difficulties establishing a stable transgenic line expressing both *per-TdT* and a pan-clock neuron driver. Relative fluorescence intensity measurements normalized to the value at t = 0 are shown separately for each clock neuron subtype. Representative single-cell traces of rhythmic cells are shown with 6^th^ order polynomial trend lines (red dotted lines). τ indicates the period. (**a**) 3 × 69-VNP expression in the LNvs (n = 11), DN1s (n = 15) and DN2s (n = 8). (**b**) PER-TDT expression in the LNvs (n = 17). (**c**) Periods of the rhythmic neurons in brain explants. Mean period ± SEM of 3 × 69-VNP was 29.27 ± 0.91 hr, PER-TDT was 28.5 ± 1.38 hr.

Next, we tested whether our new reporters can be also used for monitoring rhythms in live preparations. Cultured whole brains of adult flies have been shown to generate rhythms in luciferase reporter activity in a tissue-autonomous manner^32^,^33^,^34^. We chose to use cultured third-instar larval brains for live imaging, as larval circadian circuits are numerically simpler yet composed of fully differentiated and functional clock neurons^35^. As expected, clock neurons in cultured larval brains displayed circadian rhythms in 3 × 69-VNP and PER-TDT reporter expression, although with a longer average period (~29 hr) (Fig. 2a–c, Movies S1 and S2). Each neuron expressed varying levels of fluorescence; thus, not all the neurons were detected. Among the detected neurons, 57% of the LNvs, 50% of DN1s and 76% of DN2s showed circadian rhythms in 3 × 69-VNP levels, and 75% of the LNvs showed rhythmic PER-TDT expression. Since the long periods were likely due to the combination of the longer endogenous rhythms of the reporter flies (free-running locomotor rhythms of *1982Clk* > *mCD8*::*RFP, 3* × *69-VNP* flies: 25.1 ± 0.13 hr; *gal1118* > *mCD8*::*Venus, per-TdT* flies: 25.3 ± 0.08 hr), the 3 hr temporal resolution and culture conditions, we limited the use of brain explants to relatively short (<8 hr) fluorescent live-imaging at 1 hr intervals in subsequent experiments.

### PDF enhances CLK/CYC-mediated transcription at night

The neuropeptide PDF mediates normal circadian locomotor rhythms by affecting the synchrony, amplitude and pace of the molecular clocks^15^,^16^,^18^,^19^ and by coordinating the phase of pacemaker neuron activity^20^. PDF activates adenylate cyclase and increases cAMP levels in PDF receptor (PDFR)-expressing neurons^36^,^37^,^38^. The rise in cAMP levels activates protein kinase A (PKA) and stabilizes TIM and PER^14^,^17^, which contributes to the phase resetting and speed control of the molecular clocks. PDF also enhances neuronal activity in a cell-autonomous manner^36^,^39^,^40^. Importantly, the stabilization of TIM via PDF signalling is activity-independent; therefore, PDF/PDFR signalling at least bifurcates into the pathway controlling neuronal excitability and the one controlling PER/TIM stability^17^. However, the possible effects of PDF signalling on clock gene transcription have not been directly examined.

**Figure 3:**
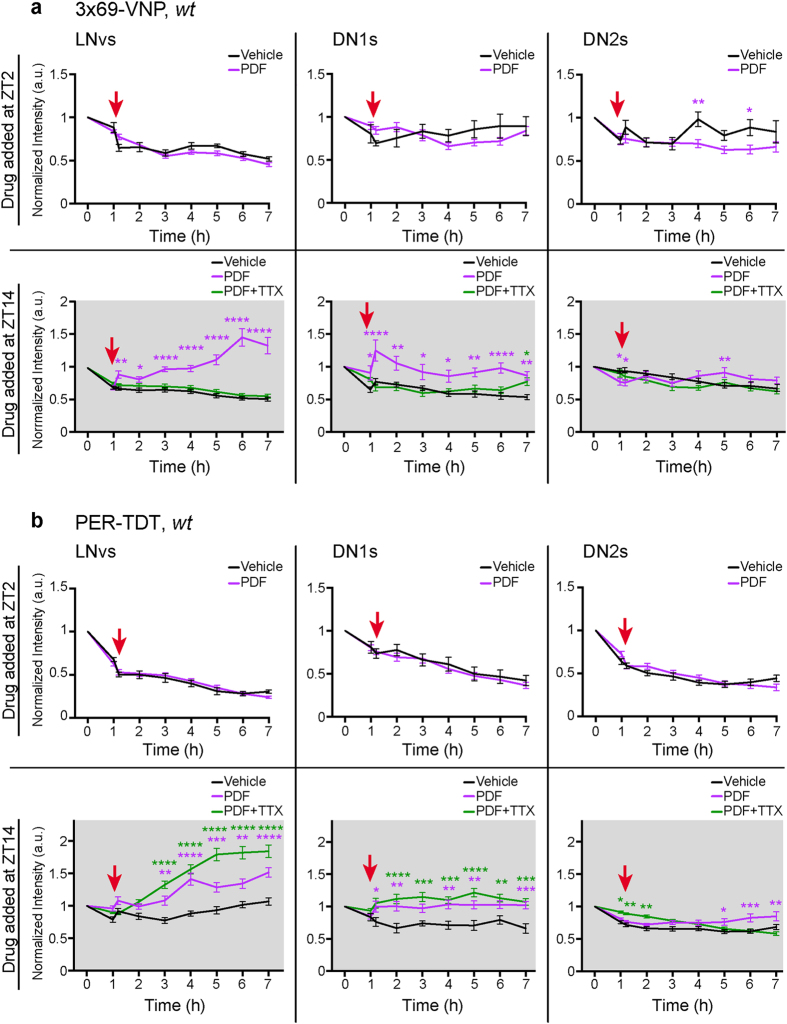
Time-of-day-dependent modulation of the molecular clockwork by PDF. LD-entrained larval brains were dissected and mounted at ZT1 or ZT13, and PDF (2 μM), DMSO or PDF (2 μM) + TTX (100 nM) was applied at ZT2 or ZT14. 3 × 69-VNP (**a**) and PER-TDT expression (**b**) in brain explants were monitored every hour by time-lapse microscopy. The mean fluorescence intensity ± SEM normalized to the value at the start of imaging (corresponding to ZT1 or ZT13) is shown. The red arrows indicate the time of drug application (ZT2 or ZT14). The colour of the asterisk indicates the group compared with the Vehicle-added group. *p < 0.05, **p < 0.01, ***p < 0.001 and ****p < 0.0001 by two-way ANOVA with Sidak’s correction for multiple comparisons. A minimum of 32 LNvs, 18 DN1s and 16 DN2s were analysed for each data point.

To better dissect the effect of PDF signalling on the clockwork, we monitored 3 × 69-VNP and PER-TDT expression in larval brain explants following a bath application of PDF. LD-entrained larvae were dissected at ZT1 or ZT13 and immediately subjected to live imaging in DD. PDF was applied at ZT2 or ZT14 (i.e., 1 hr after the start of live imaging). PDF application at ZT2 did not alter 3 × 69-VNP or PER-TDT expression in any larval clock neuron subtype. In contrast, PDF application at ZT14 upregulated 3 × 69-VNP and PER-TDT levels in the LNvs and DN1s. PDF application did not detectably affect reporter expression in the DN2s (Fig. 3a and b). Reporter expression levels continued to rise in the LNvs and DN1s during the course of imaging following the application of PDF at ZT14. This is consistent with the previous finding by Klose *et al*. that signalling downstream of PDFR continues to be active as long as PDF is present in the medium in *ex vivo* preparations^41^. As the addition of PDF had no effect on the expression profiles of 3 × 69-VNP and PER-TDT in cultures prepared from the *pdfr* hypomorphic mutant larvae, even at ZT14, the effect of PDF is specific and occurs via PDFR (Fig. S2a). Furthermore, PDF upregulated 3 × 69-VNP and PER-TDT levels at ZT14 but not at ZT2 also in *pdf01* mutants (Fig. S3). These results indicate that PDF upregulates CLK/CYC-mediated transcription and PER levels in the LNvs and DN1s at night but not during the day, and this nighttime-specific response of the molecular clock is controlled independently of the timing of PDF release.

To test whether neuronal excitability is involved in the reporter upregulation induced by PDF at ZT14, we co-applied the voltage-gated sodium channel blocker tetrodotoxin (TTX) and PDF to the brain explants. PER-TDT upregulation by PDF was insensitive to TTX (Fig. 3b). This finding parallels the observation that PDF stabilizes TIM through a mechanism independent of neuronal activity^17^. In contrast, TTX blocked the PDF-mediated upregulation of 3 × 69-VNP (Fig. 3a), indicating that PDF affects CLK/CYC-mediated transcription via time-of-day- and activity-dependent mechanisms.

**Figure 4:**
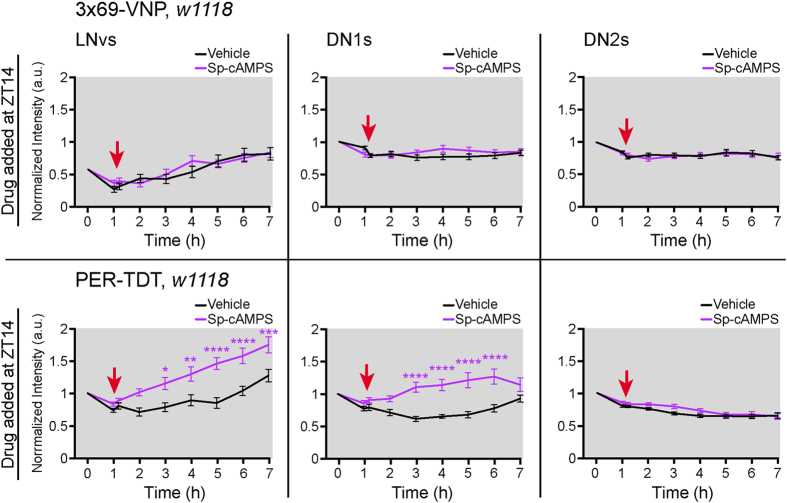
PKA activity enhances PER stability without affecting CLK/CYC-mediated transcription. LD-entrained larval brains were dissected and mounted at ZT13, and PKA activator Sp-cAMPS (150 μM) or H_2_O (vehicle) was applied at ZT14. Mean fluorescence intensities ± SEM of 3 × 69-VNP (**a**) and PER-TDT (**b**) normalized to the value at the start of imaging (ZT13) are shown. A minimum of 44 LNvs, 44 DN1s and 46 DN2s for PER-TDT and 64 LNvs, 28 DN1s and 38 DN2s for 3 × 69-VNP were analysed. The red arrows indicate the time of drug application. The colour of the asterisk indicates the group compared with the Vehicle-added group. *p < 0.05, **p < 0.01, ***p < 0.001 and ****p < 0.0001 by two-way ANOVA with Sidak’s correction for multiple comparisons.

To further test if the upregulation of PER-TDT by PDF is mediated by PKA activation^14^, we applied the cAMP analogue Sp-adenosine-3′, 5′- cyclic monophosphorothioate triethyamine (Sp-cAMPS), which specifically activates PKA^42^, to the larval brain explants at ZT14. Sp-cAMPS did not affect 3 × 69-VNP levels but significantly increased PER-TDT levels (Fig. 4), consistent with the role of cAMP-PKA signalling in PER stabilization^14^. Taken together, these results demonstrate that PDF/PDFR-signalling modulates the molecular clockwork specifically at night via two pathways: one pathway involving an increase in CLK/CYC-mediated transcription in an activity-dependent mechanism and the other involving enhancement of PER stability through cAMP-PKA activation, independent of neuronal excitability.

### Cell-autonomous, activity-dependent upregulation of clock gene transcription by PDF

TTX blocks spontaneous firing generated by cell-intrinsic mechanisms as well as the action potentials triggered by synaptic inputs^43^,^44^. To distinguish between these two possibilities, we monitored 3 × 69-VNP expression in dissociated cultured neurons in real time. Cultures were prepared from LD-entrained larvae expressing 3 × 69-VNP and the clock neuron marker (*1982clk-gal4, UAS-mCD8*::*RFP*) at approximately ZT11, then incubated for two days in DD and then subjected to time-lapse imaging starting at CT18. The cultured neurons were morphologically intact, and their neurites continued to grow and elaborate as previously shown^45^, even during the time-lapse imaging (Fig. S2b).

**Figure 5:**
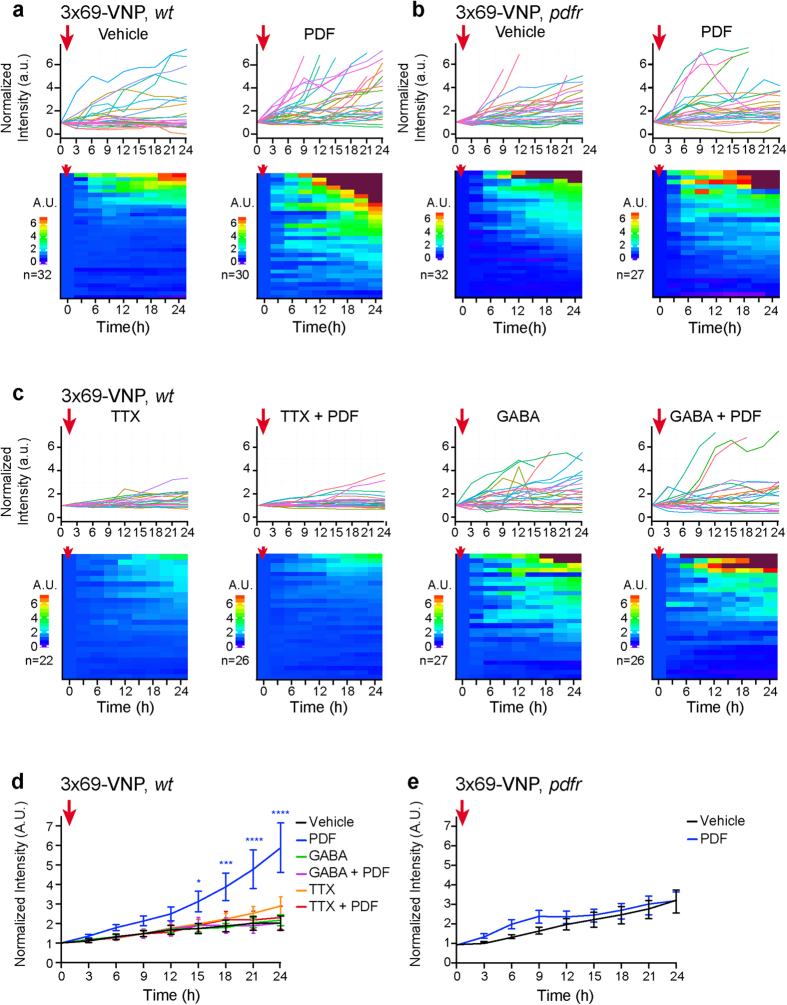
PDF cell-autonomously upregulates CLK/CYC-mediated transcription by an activity-dependent mechanism. 3 × 69-VNP expression in cultured clock neurons in response to various drug treatments. Graphs and heatmaps show the relative fluorescence intensity time course of single cells normalized to the value at t = 0. Dark red indicates values exceeding the uppermost colour scale of the heatmap. The red arrows indicate the time of drug application. (**a**) Wild-type clock neurons after the addition of PDF (20 μM) or vehicle at t = 0. (**b**) Clock neurons in *pdfr* mutants with PDF or vehicle treatment. (**c**) Wild-type clock neurons after the addition of TTX (100 nM) or GABA (10 μM), with or without PDF (20 μM) at t = 0. (**d**) Average 3 × 69-VNP fluorescence levels in wild-type clock neurons with the addition of different drugs. (**e**) Average 3 × 69-VNP levels in *pdfr* clock neurons treated with PDF or vehicle. Error bar, SEM. *p < 0.05, **p < 0.01, ***p < 0.001 and ****p < 0.0001 by two-way ANOVA with Sidak’s correction for multiple comparisons.

All of the *1982clk* > *mCD8*::*RFP* clock neuron marker-positive cells co-expressed 3 × 69-VNP; however, 3 × 69-VNP levels showed steady increases over time without detectable rhythms. Remarkably, the addition of PDF caused a significant further increase in 3 × 69-VNP levels (Movie S3, Fig. 5a and d). The addition of PDF had no significant effect on 3 × 69-VNP expression in the culture prepared from the *pdfr* mutant larvae (Fig. 5b and e), confirming that the effect of PDF *in vitro* is specific and via PDFR, as in brain culture. Furthermore, TTX or the inhibitory neurotransmitter GABA completely inhibited the upregulation of 3 × 69-VNP levels in response to acute PDF treatment (Fig. 5c and d). These results indicate that PDF upregulates CLK/CYC-mediated transcription by a cell-autonomous, activity-dependent mechanism.

## Discussion

Bioluminescence-based circadian reporters allow non-invasive, long-term recording with a high temporal resolution and have been successfully used to monitor rhythmicity in real time^32^,^33^,^34^. In contrast, fluorescent reporters can achieve the higher spatial resolution necessary to distinguish individual cells in a cluster and even to characterize intracellular protein dynamics. Using two new fluorescent reporters of the *Drosophila* molecular clock, we were able to separately interrogate two important aspects of the clockwork: CLK/CYC-mediated transcription from E-boxes and PER intracellular dynamics, both at a single-cell resolution and in real time.

**Figure 6:**
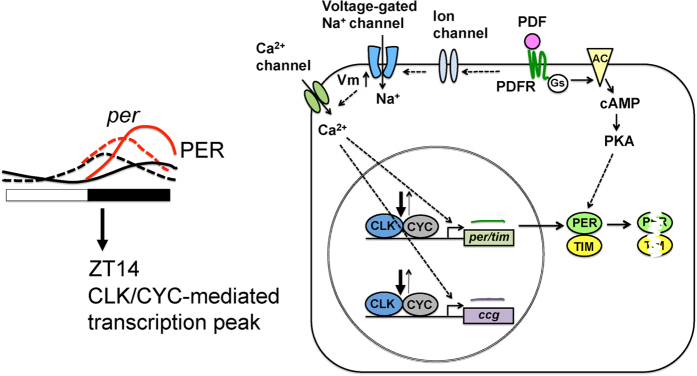
Model of the interplay between the molecular clockwork and PDF signalling. Nighttime-specific role of PDF in the LNv and DN1 clock neurons in the pacemaker circuit. At night, PDF depolarizes the membrane potential and leads to the upregulation of CLK/CYC-mediated transcription. PDF also promotes PER stability in a manner independent of neuronal activity via cAMP-PKA signalling. This nighttime-specific action of PDF on the molecular clock can amplify molecular oscillations and contribute to the synchrony of the pacemaker neurons. Dotted arrows indicate possible indirect controls.

Our study confirms and extends the mechanistic links between network interactions and molecular clockworks, particularly those mediated by PDF signalling. PDF has been shown to trigger multiple downstream effects even within the same neuron. We showed that PDF bath application upregulates PER-TDT in whole-brain cultures, consistent with previous findings indicating that PDF stabilizes PER and TIM via PKA activation independently of neuronal activity^14^,^17^. In addition, we demonstrated for the first time that PDF also enhances CLK/CYC-mediated transcription through a cell-autonomous, activity-dependent but cAMP-PKA signalling-independent mechanism. Furthermore, the effect of PDF on PER stability and clock gene transcription occurs at night but not during daytime (Figs 3,4 and 6).

PDF signalling occurs via both cAMP-dependent and cAMP-independent pathways in cockroach circadian pacemaker neurons to increase the intracellular Ca^2+^ concentration^40^. Similarly, we demonstrated here the presence of cAMP-independent pathway in *Drosophila* PDF signalling, which leads to the activation of clock gene transcription (Fig. 4a). Activity-dependent gene expression triggered by increases in Ca^2+^ concentration is a widespread phenomenon in the nervous system^46^. Although it is beyond the scope of this study to identify the mechanisms by which PDF leads to the activity-dependent (TTX-sensitive) upregulation of CLK/CYC-transcription, we speculate that Ca^2+^ influx downstream of PDF/PDFR signalling leads to an upregulation of clock gene transcription. As implied by Agrawal *et al*.^47^, PDFR-dependent increase in Ca^2+^ concentration may be mediated by IP_3_/Ca^2+^ signalling.

How does PDF confer its time-of-day-dependent effects on the molecular clock? PDF rhythmically accumulates at the s-LNv dorsal termini with a peak around ZT2^48^, and is predicted to be released at a higher rate at ZT2 than at ZT14^49^. However, our observation of ZT14-specific reporter upregulation even in the absence of endogenous PDF (Fig. S3) indicates that intracellular timing mechanisms (but not the rhythms in PDF availability) gate the molecular clock transcriptional and post-transcriptional responses to PDF. Rhythms in PDFR expression might be a possible mechanism of time-of-day-dependent PDF response, although the precise daily patterns of PDFR expression are yet to be characterized. Klose *et al*. showed that the sensitivity of the s-LNvs to PDF, measured based on the cAMP concentration, exhibits daily rhythms and peaks at dawn^41^. This suggests that cAMP signalling is permissive for the PER stabilization effect of PDF even at its nadir and the mechanisms downstream of cAMP production gate the PER stabilization effect at night.

The electrical activity of clock neurons is rhythmic and highest from the late night to early morning^49^,^50^,^51^. As intracellular Ca^2+^ levels in the s-LNvs and DN1s peak at approximately the same time^20^, rhythms in neuronal excitability and Ca^2+^ may gate the transcriptional response to PDF. We showed that TTX inhibits the nighttime-specific upregulation of clock gene transcription via PDF in brain explant cultures (Fig. 3a). These results suggest the possibility that synaptic inputs to the LNvs and DN1s control the neuronal activity relevant for gating the transcriptional response to PDF. On the other hand, PDF addition to dissociated neurons uniformly triggered TTX-sensitive transcriptional upregulation despite the lack of rhythms (Fig. 5a,c and d). This result suggests that the transcriptional upregulation induced by PDF may be the default state, i.e., that the clock may negatively regulate clock gene transcription unresponsive to PDF in the morning. As TTX can also inhibit spontaneous firing occurring independently of synaptic inputs, these results also imply that cell-autonomous control of neuronal firing rhythms by the molecular clock may be involved in the mechanisms gating the response to PDF. Fluorescent circadian reporter imaging combined with neuronal silencing or excitation by genetic tools will facilitate further studies to investigate whether the neuronal activity relevant for the response to PDF is mediated by circuit properties or by cell-autonomous mechanisms.

The nighttime-specific effect of PDF on clock gene transcription may be related to the mechanism of phase shift. Light increases the firing rate of pacemaker neurons^52^ and likely induces PDF release from the LNvs. It has also been shown that acute induction of firing mimics the effect of light in phase shift^12^. Therefore, an increase in PDF release in the early night by a light pulse when PDF levels should normally be low may trigger phase delay via enhanced clock gene transcription. Our finding that PDF-mediated activation of clock gene transcription is activity-dependent (Figs 3a and 5) also supports this possibility.

The time-of-day-dependent modulation of the molecular clockwork by PDF may also enhance the synchronization of clock neurons. CLK/CYC-transcription is active during the late day-early night and reaches a maximum around ZT14. PER maximally accumulates somewhat later, in the middle or near the end of the night^23^. As reported previously^14^, the effect of PDF on PER stabilization likely occurs in the late night to slow down the pace of intrinsically fast-paced clock neurons, such as the s-LNvs and DN1a (same as larval LNvs and DN1s)^19^. Furthermore, by stimulating CLK/CYC-mediated transcription in the early night, PDF signalling can amplify molecular clock oscillations, which contributes to phase synchrony (Fig. 6). Interestingly, a computational study predicts that VIP, the functional ortholog of PDF, can mediate synchrony across SCN neurons by inducing *per* expression only if VIP signalling occurs in-phase with *per* transcription^53^. Our results strikingly parallel this model and suggest that neuropeptide signalling in-phase with core clock gene transcription is a conserved principle for achieving pacemaker neuron synchrony.

It is noteworthy that reporter upregulation upon PDF application to brain explants was observed in the LNvs and DN1s but not in the DN2s (Figs 3 and 4). Adult DN2s express PDFR and can be activated by PDF^38^,^54^. Although larval DN2s also express PDFR^30^, the signalling downstream of PDFR in these cells may differ from that in the LNvs and DN1s. This finding underscores that PDF has multiple cell-type-specific roles.

One of our most intriguing observations is the lack of discernible circadian rhythms in 3 × 69-VNP expression in dissociated neuron cultures (Fig. 5). This finding suggests a possibility that robust transcriptional rhythms may require intact network communication and parallels the notion that circuit properties strongly influence the robustness of the mammalian SCN clocks^6^,^55^.

A recent study by Mezan *et al*. from Kadener’s group used another fluorescent circadian transcriptional reporters and showed that PDF signalling negatively regulates clock gene expression^56^. The differences in experimental setup and timescale of observations likely contributed to the differences in the conclusions between our and their studies. Whereas we focused on analysing acute response to PDF, Mezan *et al*. investigated the effect of *pdfr* mutation on *tim* transcription at steady-state or following the induction of CLK-GR transgene nuclear localization to increase CLK/CYC-mediated transcription. Before induction, CLK-GR acts dominant negatively to inhibit clock gene transcription, and it takes at least 24 hr to observe the clock gene *tim* transcriptional upregulation after CLK-GR induction. It will be interesting to compare short- and long-term effect of PDF on clock gene transcription using both experimental systems in future studies.

Cell type-specific differences in PDF signalling, downstream molecular components and relationships with neuronal activity are important issues to be addressed in future studies. There are likely other important neuropeptides and neurotransmitters that link neuronal communication with the molecular clockwork. Our new fluorescent reporters can be combined with optogenetic tools and genetically encoded calcium reporters to manipulate and analyse neuronal activity; however, the period estimated from the fluorescence time-lapse imaging could be imprecise due to its inherent lower temporal resolution compared with bioluminescence recordings. As is often the case in any *Drosophila* transgenic line, we also observed that genetic background affects the period length of the reporter lines independently of the copy number of the fluorescent reporters. Our results also suggest that culture conditions of brain explants impact the rhythmicity and period of the reporters. Given these advantages and caveats, the application of our fluorescent reporters to imaging in intact fly brains^20^,^57^ may offer powerful tools to further decipher the intricate interactions between neuronal signalling and the molecular clockwork.

## Methods

### Fly strains

*Drosophila* were reared at 25 °C on a corn-meal medium under 12 hr:12 hr light-dark (LD) cycles. The *1982clk-gal4* line was provided by N.R. Glossop^58^. The GAL4 enhancer trap line *gal1118*^59^, and *pdfr*^*5304*^ 60, *pdf*^*01*^ (Bloomington stock center, nb 26654) have been previously characterized.

*UAS-mCD8*::*VENUS* and *UAS-mCD8*::*RFP* constructs were generated by exchanging the GFP coding sequence of the *pUAST/mCD8-GFP* construct provided by L. Luo61^61^ to *Venus* cDNA or *mRFP* cDNA. The resulting constructs were introduced into the genome by P-element-mediated germline transformation.

To generate *3* × *69-VNP* flies, the *hs43* basal promoter sequence from *pCaSpeR-hs43-lacz* (GenBank accession number X81643), the *Venus-NLS-PEST1* coding sequence^24^ and 1 kb of the *per* 3′ UTR were PCR-amplified and ligated into the vector containing three tandem copies of the 69-bp CRS of *per* (pCaSpeR-*per69* × *3-luc*), thus replacing the luciferase coding sequence and the SV40 3′ UTR^26^. The resulting *pCaSpeR-3* × *69-Venus-UTR* vector was used for P-element-mediated germline transformation.

To generate the *per-TdT* transgenic line, the *pStinger* vector was modified by removing the coding sequences of 5xUAS and EGFP-NLS and adding the *attB* sequence between the P-element 3′ end and a *gypsy* insulator. A 13.2 kb *per* genomic fragment (from the *BamHI* site at −4.2 kb to the *EcoRI* site located ca. 2 kb downstream of exon 8) was cloned into a modified *pStinger* vector. The tdTomato (TdT) coding sequence, amplified from the *pRSET-B/tdTomato* plasmid (generated by R. Tsien and provided by L. Luo), and a 1 kb *per* 3′UTR sequence were cloned into the *pBS* plasmid. A *BamHI*-*PsiI* fragment from this plasmid containing the *TdT-3*′*UTR* was then ligated to the *BamHI*-*PsiI* fragment of *pStinger-per13.2*, which resulted in the *per* (exon 1–5)-*TdT*-*3*′*UTR* fusion construct (*per-TdT* vector). The *per-TdT* strains were generated by integrating the *per-TdT* vector into the attP16 (2R) and attP40 (2L) landing sites by PhiC13-mediated site-specific integration. Injections were performed by BestGene Inc. The *per-TdT (attP16*) and *per-TdT (attP40*) lines were crossed to recombine both transgenes on the 2^nd^ chromosome.

### Larval brain culture

The brains of LD-entrained non-wandering L3 larvae were dissected in ice-cold saline solution^62^ at ZT11. The imaginal discs were left intact to prevent any tearing of the tissue. Dissected brains were kept on ice in modified Schneider’s medium (SM^active^)^63^ supplemented with 5 mM Bis-Tris (Sigma). For *ex vivo* brain culture, the brain explants were mounted on a glass-bottom dish (35 mm MatTek petri dish, 20 mm microwell with 0.16/0.19 mm coverglass) in a fibrinogen clot prepared by adding thrombin (bovine thrombin, Sigma) to fibrinogen (bovine fibrinogen, Calbiochem) as previously described^64^. The glass-bottom well was filled with the SM^active^ medium and covered by a Teflon membrane permeable to oxygen. The cultured brains were kept at 25 °C in 80% relative humidity and in the dark for a few hours prior to time-lapse imaging to allow the brains to set. Time-lapse imaging was performed in the same culture conditions, with images acquired every 3 hr.

For pharmacological experiments on brain explants, brains were dissected either at ZT1 or at ZT13 and cultured in dissecting saline solution (equivalent to hemolymph-like saline) without a Teflon membrane. The brain explants were immediately imaged to establish a baseline. Approximately 1 hr after the start of the time-lapse imaging, the neuropeptide PDF (custom-made by Chi Scientific, H-NSELINSLLSLPKNMNDA-OH; 2 μM) was added alone or in combination with 100 nM TTX (Cayman Chemical). For the control condition, the vehicle (DMSO) was added. Sp-cAMPS (sc-201571 from Santa Cruz Biotechnology, 150 μM) was added alone and the vehicle (H_2_O) was used as a control. Time-lapse imaging was performed in the same conditions as described above with images acquired every hour for 8 hr.

### Primary neuron culture

Dissociated neuron culture was performed as previously described^45^,^64^ with the following modifications. In brief, the dissected brains were enzymatically treated with 50 units/mL papain (Worthington) and mechanically dissociated. The cell suspension was then plated on glass-bottom dishes (35 mm MatTek petri dish, 10 mm microwell with 0.16/0.19 mm coverglass) coated with concanavalin A (Sigma). Once the cells were attached to the glass bottom, the dish was flooded with SM^active^. The dissociated neuron culture was incubated at 25 °C in 80% relative humidity and constant darkness for 2 days prior to time-lapse imaging. For pharmacological experiments, 2 or 20 μM PDF, 100 nM TTX, 10 μM GABA (Sigma), or the vehicle (DMSO or ddH_2_O) was added to the cell culture medium just before the start of the time-lapse imaging. Time-lapse experiments were conducted in the same culture conditions, and the images were captured every 3 hr.

### Microscopy and image analysis

A Leica TCS SP5 tandem scanner confocal microscope was used for fluorescence imaging. The same parameter settings were used to image all samples of the same type (dissected brains, cultured brains or cultured neurons). Freshly dissected larval and adult brains were scanned using a 40x water-immersion objective with the galvo scanner at 400 Hz. For time-lapse imaging, 20x, 40x or 63x objectives were used, and the images were acquired using the resonant scanner at 8,000 Hz with high-sensitivity HyD detectors. A bi-directional scan was used together with an 8x line average. Z-section steps of 1.7 μm × 8 and 2 μm × ~40 were used for imaging the dissociated cultured neurons and *ex vivo* brain culture, respectively. A 514-nm laser was used to excite the VENUS fluorophore (0.68 μW/cm^2^ to image VNP, 0.40 μW/cm^2^ for mCD8::VENUS), and a 561-nm laser was used for the TdTomato and mRFP fluorophores (14.8 μW/cm^2^ for PER-TDT, 5.71 μW/cm^2^ for mCD8::RFP). The laser intensity was measured at the level of the sample with a microscope slide power meter (Thorlabs, S170C).

The raw data of the larval and adult brain images taken at different time points and the time-lapse movies of the *ex vivo* brain cultures were analysed with FIJI software^65^. Briefly, a SUM-stack containing the clock neuron cluster of interest was constructed, and the area of each neuron was manually determined. The mean fluorescence intensity of the defined area in the SUM-stack was measured. Three nearby areas were also measured to analyse the background fluorescence level. The corrected total relative intensity of each cell was calculated as follows:


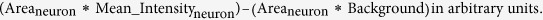


The fluorescence intensities in the time-lapse movies of the cultured neurons were measured using Imaris software (Bitplane). A 3D mask (region of interest in 3D) was built for each cell by thresholding the 3 × 69-VNP or PER-TDT fluorescence levels after background subtraction. The intensity SUM in each 3D mask was then extracted from the statistical data that were automatically generated by the program. For presentation purposes only, some of the images and time-lapse movies were processed with a 10x iterative deconvolution using AutoQuant (MediaCybernetics) and Imaris. When necessary, time-lapse images were also treated for 3D correction drift using the ImageJ plugin (NIH).

### Time series data analysis

Fluorescence-intensity time-series data were normalized to the value at t = 0. Heatmaps representing the fluorescence-intensity time course were generated with an in-house R script. Each row represents the data of a single cell. Rows were ordered by the highest intensity over 24 hr.

For rhythm analysis, a combination of manual inspection and Maximum Entropy Spectral Analysis (MESA) was used to detect rhythmicity and estimate the period. MESA was chosen because, unlike other methods, it is adapted for the detection of rhythms in short or noisy time series^66^. First, time course plots of the intensity were generated using Excel or Prism (GraphPad Prism version 6.0c for Mac, GraphPad Software, San Diego, California, USA, www.graphpad.com). Because we found that 6^th^-order polynomial regression models best fit the data, we superimposed the 6^th^-order polynomial trend lines on the graphs to facilitate the detection of rhythmicity. The time-series data with circadian rhythms, defined by a peak-to-peak interval from 18 to 36 hr, were then identified by manual inspection. However, the polynomial trend lines were not used to determine the period. In parallel, the intensity time-series data without normalization were analysed with MESA without filtering. The time-series data were scored as circadian only when both the manual and MESA analyses detected the rhythms with a period between 18 and 36 hr.

### Statistical analysis

Statistical analyses were performed with GraphPad Prism software. To compare the effects of the drug treatments in the pharmacological experiments (Figs 3–5), a two-way ANOVA with a Sidak test to correct for multiple comparisons was used.

## Additional Information

**How to cite this article**: Sabado, V. *et al*. Fluorescence circadian imaging reveals a PDF-dependent transcriptional regulation of the *Drosophila* molecular clock. *Sci. Rep.* **7**, 41560; doi: 10.1038/srep41560 (2017).

**Publisher's note:** Springer Nature remains neutral with regard to jurisdictional claims in published maps and institutional affiliations.

## Supplementary Material

Supplementary Information

Supplementary Movie S1

Supplementary Movie S2

Supplementary Movie S3
