# Supplementary material for: Fluorescence circadian imaging reveals a PDF-dependent transcriptional regulation of the Drosophila molecular clock
Source: Sci Rep. 2017 Jan 30;7:41560. doi: 10.1038/srep41560 (PMC5278502; doi:10.1038/srep41560)
Supplement: Supplementary Information [file srep41560-s1.pdf]

Supplementary information

Fluorescence circadian imaging reveals a PDF-dependent transcriptional regulation of the *Drosophila* molecular clock

Virginie Sabado, Ludovic Vienne, José Manuel Nunes, Michael Rosbash, and Emi Nagoshi

Inventory of supplemental information

1. Supplemental Figures S1- S3
2. Captions of the supplemental movie S1-S3

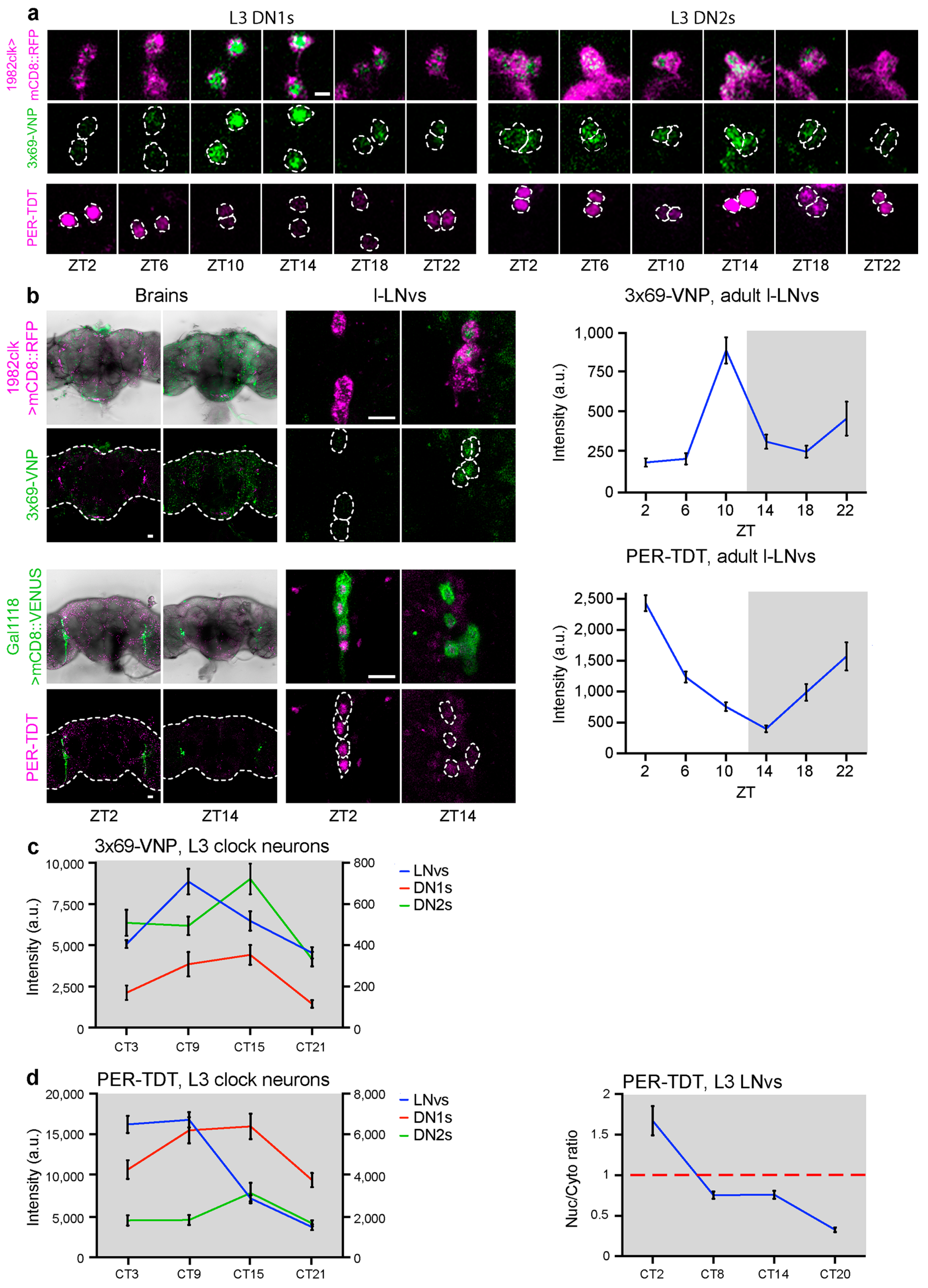
a
L3 DN1s
L3 DN2s
1982clk> mCD8::RFP
3x69-VNP
PER-TDT
ZT2
ZT6
ZT10
ZT14
ZT18
ZT22
b
Brains
l-LNvs
3x69-VNP, adult l-LNvs
1982clk >mCD8::RFP
Gal1118 >mCD8::VENUS
Intensity (a.u.)
ZT
PER-TDT, adult l-LNvs
c
3x69-VNP, L3 clock neurons
LNvs
DN1s
DN2s
CT3
CT9
CT15
CT21
d
PER-TDT, L3 clock neurons
PER-TDT, L3 LNvs
Nuc/Cyto ratio
CT2
CT8
CT14
CT20

Figure S1. Fluorescent reporters of transcriptional and PER protein rhythms. (a) Around-the-clock expression of 3x69-VNP (top panels) and PER-TDT (bottom) in larval DN1s and DN2s. In *3x69-VNP* flies, DNs were labelled with *1982clk-gal4*, *UAS-mCD8::RFP*. In *per-TdT* flies, DNs were identified by anatomical characteristics without cell markers. Scale bar, 5 µm. (b) 3x69-VNP and PER-TDT expression in adult brains. Left panels show representative confocal images of whole brains and the l-LNvs labelled with *1982clk-gal4*, *UAS-mCD8::RFP* or *gal1118*, *UAS-mCD8::Venus* at 2 time points in LD. Scale bar, 20 µm. Right panels, around-the-clock quantification of the reporter fluorescence levels in adult l-LNvs. Mean ± SEM. A minimum of 30 neurons were analysed at each time point. (c and d) 3x69-VNP (c) and PER-TDT (d) expression levels in all clock neurons and PER-TDT nuclear/cytoplasmic ratio in larval LNvs (d) in DD2 analysed every 6 hr. Mean ± SEM. A minimum of 26 neurons were analysed at each time point.

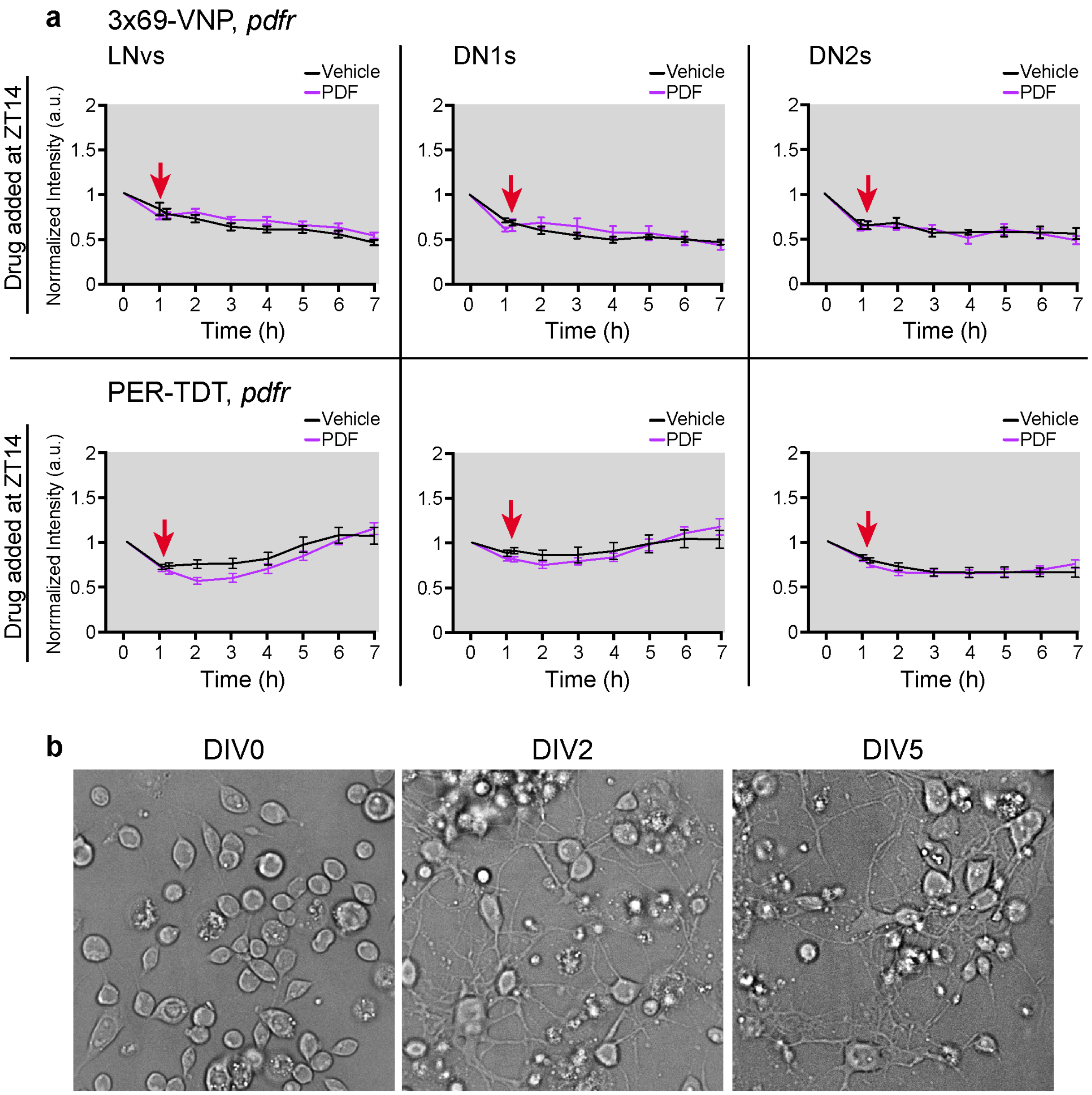

Figure S2. Controls for pharmacological experiments with brain explants and dissociated cultured neurons.
(a) 3x69-VNP and PER-TDT fluorescence levels in the clock neurons of *pdfr* mutant brain explants. LD-entrained larval brains were dissected and mounted at ZT13, and PDF (2 μM) or DMSO (vehicle) was applied at ZT14. A minimum of 36 LNvs, 12 DN1s and 10 DN2s were analysed. No significant differences between two groups by two-way ANOVA with Sidak's correction for multiple comparisons. (b) Bright-field images of the cultured neurons on the day the neurons were plated (day 0 *in vitro*, DIV0) and at DIV2 and DIV5. The snapshots at DIV2 and DIV5 were taken during time-lapse imaging. Extensive neurite growth was observed from DIV2 to DIV5.

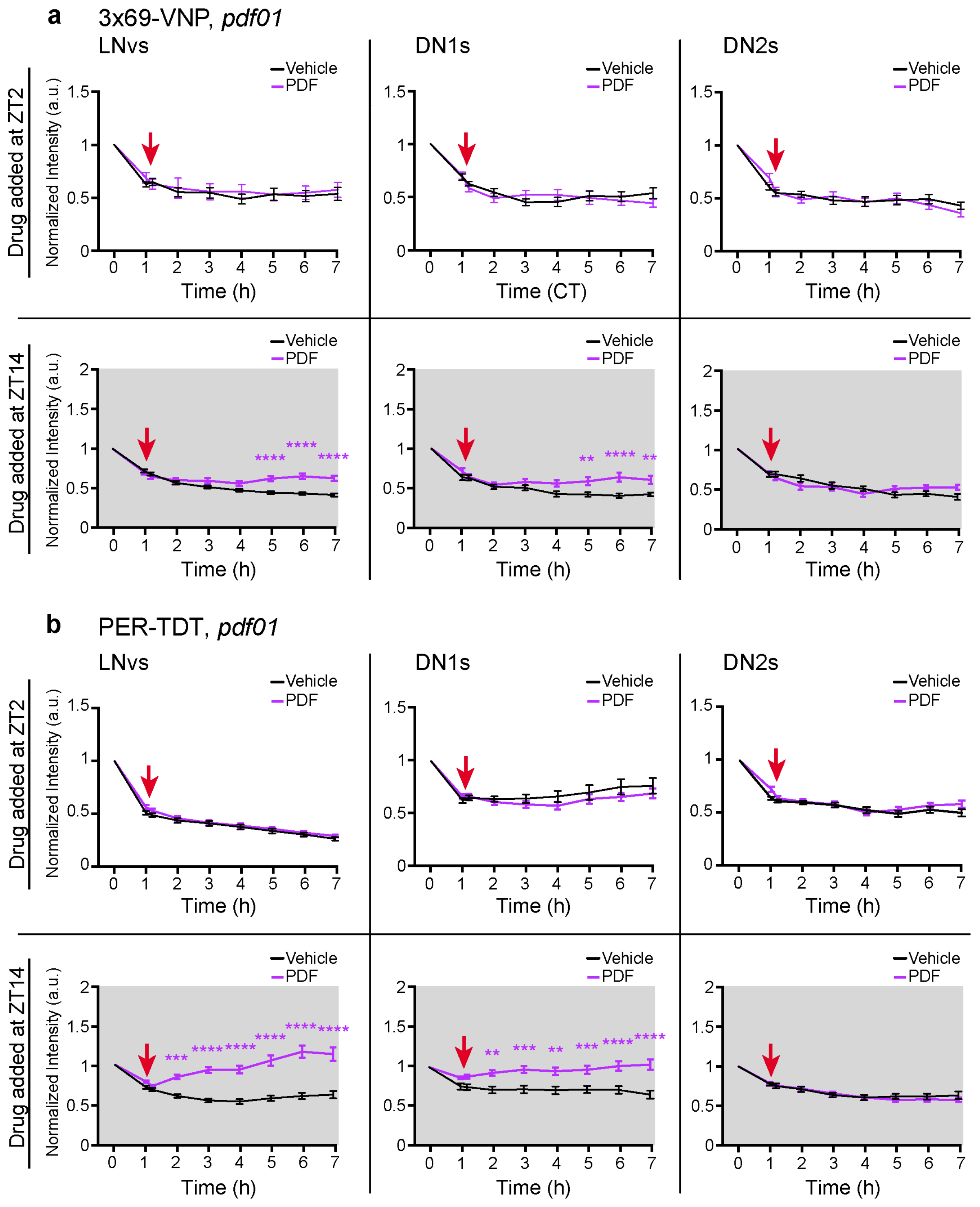

Figure S3. Time-of-day-dependent modulation of the molecular clockwork is independent of the release of endogenous PDF.
3x69-VNP (a) and PER-TDT (b) fluorescence levels in the clock neurons of *pdf01* mutant brain explants. LD-entrained larval brains were dissected and mounted at ZT1 or ZT13, and PDF (2 μM) or DMSO (vehicle) was applied at ZT2 or ZT14. A minimum of 60 LNvs, 30 DN1s and 42 DN2s for PER-TDT and 60 LNvs, 26 DN1s and 24 DN2s for 3x69-VNP were analysed. The colour of the asterisk indicates the group compared with the Vehicle-added group. *$p<0.05$, **$p<0.01$ by two-way ANOVA with Sidak's correction for multiple comparisons.

Captions of the supplemental movie S1-S3

Movie S1

3x69-VNP expression in a larval brain explant. Images were taken every 3 hr for 54 hr. Left, clock neurons expressing *1982clk-gal4*, *UAS-mCD8::RFP* are shown in magenta, and 3x69-VNP is shown in green. Right, 3x69-VNP levels are shown as a blue gradient, in which lighter blue indicates higher VNP expression.

Movie S2

PER-TDT expression in a larval brain explant. Images were taken every 3 hr for 48 hr. Left, PER-TDT is shown in magenta. The LNvs are labelled with *mCD8::Venus* driven by *gal1118* (green). Note that many glial cells, mainly located on the surface of the brain, start to express both PER and PER-TDT in the late third instar larval brain. The arrow indicates the position of the LNvs. Middle, TDT fluorescence levels are converted to a blue gradient. Right, to evaluate fluorescence levels in the neurons without the overlapping signals from glial cells, the LNvs are magnified and shown as a 3D surface-rendered movie. PER-TDT fluorescence levels are shown with a rainbow scale.

Movie S3

3x69-VNP expression in cultured clock neurons with PDF or vehicle treatment at t=0. Clock neurons are labelled with *1982clk*-gal4, *UAS-mCD8::RFP* (Left: vehicle, Right: +PDF). Images were taken every 3 hr for 24 hr. Top panels, 3x69-VNP is shown in green, and the clock neuron marker is shown in magenta. Bottom, VNP fluorescence is converted to pseudocolour on a rainbow scale, with red corresponding to the highest level.
